# Supplementary material for: HIVprotI: an integrated web based platform for prediction and design of HIV proteins inhibitors
Source: J Cheminform. 2018 Mar 9;10:12. doi: 10.1186/s13321-018-0266-y (PMC5845081; doi:10.1186/s13321-018-0266-y)
Supplement: Supplementary file 2 — Additional file 2. Source code of HIVProtI web server. [file 13321_2018_266_MOESM2_ESM.zip › HIVprotI_Source-code/hivprotI/weka-3-6-6/doc/allclasses-frame.html]

All Classes


**All Classes**
  

AbstractAssociator
  
AbstractClusterer
  
AbstractDataSink
  
AbstractDataSinkBeanInfo
  
AbstractDataSource
  
AbstractDataSourceBeanInfo
  
AbstractDensityBasedClusterer
  
AbstractEvaluator
  
AbstractFileLoader
  
AbstractFileSaver
  
AbstractLoader
  
AbstractSaver
  
AbstractStringDistanceFunction
  
AbstractTestSetProducer
  
AbstractTestSetProducerBeanInfo
  
AbstractTimeSeries
  
AbstractTrainAndTestSetProducer
  
AbstractTrainAndTestSetProducerBeanInfo
  
AbstractTrainingSetProducer
  
AbstractTrainingSetProducerBeanInfo
  
AdaBoostM1
  
Add
  
AddClassification
  
AddCluster
  
AddExpression
  
AddID
  
*AdditionalMeasureProducer*
  
AdditiveRegression
  
AddNoise
  
AddValues
  
ADNode
  
ADTree
  
Agrawal
  
AlgorithmListPanel
  
AlgVector
  
AllFilter
  
AllJavadoc
  
AlphabeticTokenizer
  
AODE
  
AODEsr
  
Apriori
  
AprioriItemSet
  
ArffLoader
  
ArffLoader.ArffReader
  
ArffPanel
  
ArffSaver
  
ArffSortedTableModel
  
ArffTable
  
ArffTableCellRenderer
  
ArffTableModel
  
ArffViewer
  
ArffViewerMainPanel
  
ASEvaluation
  
ASSearch
  
AssociationsPanel
  
*Associator*
  
Associator
  
AssociatorBeanInfo
  
AssociatorCustomizer
  
AssociatorEvaluation
  
Attribute
  
*AttributeEvaluator*
  
AttributeExpression
  
AttributeListPanel
  
AttributeLocator
  
AttributePanel
  
AttributePanelEvent
  
*AttributePanelListener*
  
AttributeSelectedClassifier
  
AttributeSelection
  
AttributeSelection
  
AttributeSelectionPanel
  
AttributeSelectionPanel
  
AttributeSetEvaluator
  
AttributeStats
  
AttributeSummarizer
  
AttributeSummarizerBeanInfo
  
AttributeSummaryPanel
  
*AttributeTransformer*
  
AttributeValueLiteral
  
AttributeVisualizationPanel
  
AveragingResultProducer
  
Bagging
  
BallNode
  
BallSplitter
  
BallTree
  
BallTreeConstructor
  
BatchClassifierEvent
  
*BatchClassifierListener*
  
BatchClustererEvent
  
*BatchClustererListener*
  
*BatchConverter*
  
BayesianLogisticRegression
  
BayesNet
  
BayesNet
  
BayesNetEstimator
  
BayesNetGenerator
  
*BeanCommon*
  
BeanConnection
  
BeanInstance
  
BeanVisual
  
BestFirst
  
BFTree
  
BIFFormatException
  
BIFParser
  
BIFReader
  
BinarySparseInstance
  
BinC45ModelSelection
  
BinC45Split
  
BIRCHCluster
  
BMAEstimator
  
BMPWriter
  
Body
  
BottomUpConstructor
  
BoundaryPanel
  
BoundaryPanelDistributed
  
BoundaryVisualizer
  
BrowserHelper
  
BuiltInArithmetic
  
BuiltInMath
  
BuiltInString
  
BVDecompose
  
BVDecomposeSegCVSub
  
C45Loader
  
C45ModelSelection
  
C45PruneableClassifierTree
  
C45PruneableClassifierTreeG
  
C45PruneableDecList
  
C45Saver
  
C45Split
  
CachedKernel
  
Capabilities
  
Capabilities.Capability
  
*CapabilitiesHandler*
  
CaRuleGeneration
  
*CARuleMiner*
  
Center
  
CfsSubsetEval
  
ChangeDateFormat
  
CharacterDelimitedTokenizer
  
ChartEvent
  
*ChartListener*
  
ChebyshevDistance
  
Check
  
CheckAssociator
  
CheckAttributeSelection
  
CheckBoxList
  
CheckClassifier
  
CheckClusterer
  
CheckEstimator
  
CheckEstimator.AttrTypes
  
CheckEstimator.EstTypes
  
CheckGOE
  
CheckKernel
  
CheckOptionHandler
  
CheckScheme
  
CheckScheme.PostProcessor
  
CheckSource
  
CheckSource
  
ChisqMixture
  
ChiSquaredAttributeEval
  
CholeskyDecomposition
  
CISearchAlgorithm
  
CitationKNN
  
ClassAssigner
  
ClassAssigner
  
ClassAssignerBeanInfo
  
ClassAssignerCustomizer
  
ClassBalancedND
  
ClassDiscovery
  
ClassDiscovery.StringCompare
  
ClassificationGenerator
  
ClassificationViaClustering
  
ClassificationViaRegression
  
Classifier
  
Classifier
  
ClassifierBeanInfo
  
ClassifierCustomizer
  
ClassifierDecList
  
ClassifierPanel
  
ClassifierPerformanceEvaluator
  
ClassifierPerformanceEvaluatorBeanInfo
  
ClassifierSplitEvaluator
  
ClassifierSplitModel
  
ClassifierSubsetEval
  
ClassifierTree
  
ClassloaderUtil
  
ClassOrder
  
ClassPanel
  
ClassValuePicker
  
ClassValuePickerBeanInfo
  
ClassValuePickerCustomizer
  
CLOPE
  
ClusterDefinition
  
*Clusterer*
  
Clusterer
  
ClustererBeanInfo
  
ClustererCustomizer
  
ClustererPanel
  
ClustererPerformanceEvaluator
  
ClustererPerformanceEvaluatorBeanInfo
  
ClusterEvaluation
  
ClusterGenerator
  
ClusterMembership
  
Cobweb
  
Colors
  
ComplementNaiveBayes
  
ComponentHelper
  
*Compute*
  
*ConditionalEstimator*
  
ConfusionMatrix
  
ConjunctiveRule
  
ConnectionEvent
  
*ConnectionListener*
  
*ConnectionNotificationConsumer*
  
ConnectionPanel
  
ConsistencySubsetEval
  
ConsoleLogger
  
Constant
  
ContingencyTables
  
ConverterFileChooser
  
ConverterUtils
  
ConverterUtils.DataSink
  
ConverterUtils.DataSource
  
Copy
  
*Copyable*
  
Copyright
  
CorrelationSplitInfo
  
CostBenefitAnalysis
  
CostBenefitAnalysisBeanInfo
  
CostCurve
  
CostMatrix
  
CostMatrixEditor
  
CostSensitiveASEvaluation
  
CostSensitiveAttributeEval
  
CostSensitiveClassifier
  
CostSensitiveClassifierSplitEvaluator
  
CostSensitiveSubsetEval
  
CoverTree
  
CrossValidationFoldMaker
  
CrossValidationFoldMakerBeanInfo
  
CrossValidationFoldMakerCustomizer
  
CrossValidationResultProducer
  
CSVLoader
  
CSVResultListener
  
CSVSaver
  
*CustomizerCloseRequester*
  
*CustomizerClosingListener*
  
*CustomPanelSupplier*
  
CVParameterSelection
  
Dagging
  
*Database*
  
DatabaseConnection
  
DatabaseConnectionDialog
  
*DatabaseConverter*
  
DatabaseLoader
  
DatabaseResultListener
  
DatabaseResultProducer
  
DatabaseSaver
  
DatabaseUtils
  
*DataFormatListener*
  
DataGenerator
  
*DataGenerator*
  
DataGeneratorPanel
  
DataNearBalancedND
  
*DataObject*
  
DataSetEvent
  
DatasetListPanel
  
*DataSink*
  
*DataSource*
  
*DataSourceListener*
  
DataVisualizer
  
DataVisualizerBeanInfo
  
DBScan
  
DbUtils
  
DDConditionalEstimator
  
Debug
  
Debug.Clock
  
Debug.DBO
  
Debug.Log
  
Debug.Random
  
Debug.SimpleLog
  
Debug.Timestamp
  
DecisionStump
  
DecisionTable
  
DecisionTableHashKey
  
Decorate
  
DefineFunction
  
*DensityBasedClusterer*
  
DensityBasedClustererSplitEvaluator
  
DerivedFieldMetaInfo
  
DiscreteEstimator
  
DiscreteEstimatorBayes
  
DiscreteEstimatorFullBayes
  
DiscreteFunction
  
Discretize
  
Discretize
  
Discretize
  
*DistanceFunction*
  
DistributeExperimentPanel
  
Distribution
  
DKConditionalEstimator
  
DMNBtext
  
DNConditionalEstimator
  
DotParser
  
DoubleVector
  
*Drawable*
  
DTNB
  
Edge
  
EditableBayesNet
  
EditDistance
  
EigenvalueDecomposition
  
Element
  
EM
  
END
  
EntropyBasedSplitCrit
  
EntropySplitCrit
  
Environment
  
*EnvironmentHandler*
  
EpsilonRange\_ListElement
  
*ErrorBasedMeritEvaluator*
  
Estimator
  
EstimatorUtils
  
EuclideanDistance
  
EuclidianDataObject
  
Evaluation
  
EvaluationUtils
  
*EventConstraints*
  
ExhaustiveSearch
  
Experiment
  
Experimenter
  
ExperimenterDefaults
  
Explorer
  
Explorer.CapabilitiesFilterChangeEvent
  
*Explorer.CapabilitiesFilterChangeListener*
  
*Explorer.ExplorerPanel*
  
*Explorer.LogHandler*
  
ExplorerDefaults
  
ExponentialFormat
  
Expression
  
Expression
  
ExtensionFileFilter
  
FarthestFirst
  
FastVector
  
FieldMetaInfo
  
FieldMetaInfo.Interval
  
FieldMetaInfo.Interval.Closure
  
FieldMetaInfo.Optype
  
FieldMetaInfo.Value
  
FieldMetaInfo.Value.Property
  
FieldRef
  
FileEditor
  
FileLogger
  
*FileSourcedConverter*
  
Filter
  
Filter
  
FilterBeanInfo
  
FilterCustomizer
  
FilteredAssociator
  
FilteredAttributeEval
  
FilteredClassifier
  
FilteredClusterer
  
FilteredSubsetEval
  
FindWithCapabilities
  
FirstOrder
  
FlexibleDecimalFormat
  
FloatingPointFormat
  
FlowRunner
  
FlowRunner.SimpleLogger
  
FPGrowth
  
FPGrowth.AssociationRule
  
FPGrowth.AssociationRule.METRIC\_TYPE
  
FPGrowth.BinaryItem
  
FromFile
  
FT
  
FTInnerNode
  
FTLeavesNode
  
FTNode
  
FTtree
  
Function
  
GainRatioAttributeEval
  
GainRatioSplitCrit
  
GaussianPriorImpl
  
GaussianProcesses
  
GeneralizedSequentialPatterns
  
GeneralRegression
  
GeneratorPropertyIteratorPanel
  
GenericArrayEditor
  
GenericObjectEditor
  
GenericPropertiesCreator
  
GeneticSearch
  
GeneticSearch
  
GeneticSearch
  
GlobalInfoJavadoc
  
GlobalScoreSearchAlgorithm
  
Grading
  
GraftSplit
  
*GraphConstants*
  
GraphEdge
  
GraphEvent
  
*GraphListener*
  
GraphNode
  
GraphPanel
  
GraphViewer
  
GraphViewerBeanInfo
  
GraphVisualizer
  
GreedyStepwise
  
GridSearch
  
GUI
  
GUIChooser
  
GUIChooser.ChildFrameSDI
  
Head
  
HierarchicalBCEngine
  
HierarchicalClusterer
  
HierarchyPropertyParser
  
HierarchyVisualizer
  
HillClimber
  
HillClimber
  
HistoryChangedEvent
  
*HistoryChangedListener*
  
HNB
  
HoldOutSubsetEvaluator
  
HostListPanel
  
HyperPipes
  
IB1
  
IBk
  
ICSSearchAlgorithm
  
Id3
  
Impurity
  
IncrementalClassifierEvaluator
  
IncrementalClassifierEvaluatorBeanInfo
  
IncrementalClassifierEvaluatorCustomizer
  
IncrementalClassifierEvent
  
*IncrementalClassifierListener*
  
*IncrementalConverter*
  
*IncrementalEstimator*
  
IndividualInstance
  
IndividualInstances
  
IndividualLiteral
  
InfoGainAttributeEval
  
InfoGainSplitCrit
  
InfoPanel
  
InfoPanelCellRenderer
  
Instance
  
InstanceComparator
  
InstanceCounter
  
InstanceEvent
  
InstanceEvent
  
InstanceJoiner
  
*InstanceListener*
  
*InstanceListener*
  
InstanceLoader
  
*InstanceProducer*
  
InstanceQuery
  
Instances
  
InstanceSavePanel
  
InstancesResultListener
  
InstancesSummaryPanel
  
InstanceStreamToBatchMaker
  
InstanceStreamToBatchMakerBeanInfo
  
InstanceTable
  
InstanceViewer
  
InterquartileRange
  
*IntervalEstimator*
  
IntVector
  
IsotonicRegression
  
ItemSet
  
IteratedLovinsStemmer
  
IteratedSingleClassifierEnhancer
  
*IterativeClassifier*
  
J48
  
J48graft
  
Javadoc
  
JComponentWriter
  
JListHelper
  
JPEGWriter
  
JRip
  
JTableHelper
  
Jython
  
*JythonObject*
  
*JythonSerializableObject*
  
K2
  
K2
  
KDConditionalEstimator
  
KDDataGenerator
  
KDTree
  
KDTreeNode
  
KDTreeNodeSplitter
  
Kernel
  
KernelEstimator
  
KernelEvaluation
  
KernelFilter
  
KKConditionalEstimator
  
KMeansInpiredMethod
  
KnowledgeFlow
  
KnowledgeFlowApp
  
KOML
  
KStar
  
KStarCache
  
*KStarConstants*
  
KStarNominalAttribute
  
KStarNumericAttribute
  
KStarWrapper
  
LabeledItemSet
  
LADTree
  
LAGDHillClimber
  
LaplacePriorImpl
  
LatentSemanticAnalysis
  
LayoutCompleteEvent
  
*LayoutCompleteEventListener*
  
*LayoutEngine*
  
LBR
  
LearningRateResultProducer
  
LeastMedSq
  
LED24
  
LegendPanel
  
LFSMethods
  
LibLINEAR
  
LibSVM
  
LibSVMLoader
  
LibSVMSaver
  
LinearForwardSelection
  
LinearNNSearch
  
LinearRegression
  
LinearRegression
  
LinearUnit
  
ListOptions
  
ListSelectorDialog
  
Literal
  
LiteralSet
  
LMT
  
LMTNode
  
*Loader*
  
Loader
  
Loader
  
LoaderBeanInfo
  
LoaderCustomizer
  
LocalScoreSearchAlgorithm
  
Logger
  
*Logger*
  
Logger.Level
  
Logistic
  
LogisticBase
  
LogitBoost
  
LogPanel
  
LogPanel
  
LogWindow
  
*LogWriter*
  
LookAndFeel
  
LovinsStemmer
  
LUDecomposition
  
LWL
  
M5Base
  
M5P
  
M5Rules
  
MahalanobisEstimator
  
Main
  
Main.BackgroundDesktopPane
  
Main.ChildFrameMDI
  
Main.ChildFrameSDI
  
*MainMenuExtension*
  
MakeDecList
  
MakeDensityBasedClusterer
  
MakeIndicator
  
ManhattanDataObject
  
ManhattanDistance
  
MappingInfo
  
MarginCalculator
  
MarginCurve
  
*Matchable*
  
MathematicalExpression
  
MathExpression
  
Maths
  
Matrix
  
Matrix
  
MatrixPanel
  
MDD
  
MedianDistanceFromArbitraryPoint
  
MedianOfWidestDimension
  
MedianOfWidestDimension
  
Memory
  
MemoryUsagePanel
  
MergeTwoValues
  
Messages
  
Messages
  
Messages
  
Messages
  
Messages
  
Messages
  
Messages
  
Messages
  
Messages
  
Messages
  
Messages
  
Messages
  
Messages
  
Messages
  
Messages
  
Messages
  
MetaBean
  
MetaCost
  
MethodHandler
  
MexicanHat
  
MIBoost
  
MIDD
  
MiddleOutConstructor
  
MidPointOfWidestDimension
  
MIEMDD
  
MILR
  
MiningFieldMetaInfo
  
MiningSchema
  
MINND
  
MIOptimalBall
  
MIPolyKernel
  
MIRBFKernel
  
MISMO
  
MISVM
  
MIWrapper
  
MixtureDistribution
  
ModelPerformanceChart
  
ModelPerformanceChartBeanInfo
  
ModelSelection
  
MultiBoostAB
  
MultiClassClassifier
  
MultiFilter
  
*MultiInstanceCapabilitiesHandler*
  
MultiInstanceToPropositional
  
MultilayerPerceptron
  
MultiNomialBMAEstimator
  
MultipleClassifiersCombiner
  
MultiScheme
  
NaiveBayes
  
NaiveBayes
  
NaiveBayesMultinomial
  
NaiveBayesMultinomialUpdateable
  
NaiveBayesSimple
  
NaiveBayesUpdateable
  
NamedColor
  
NBTree
  
NBTreeClassifierTree
  
NBTreeModelSelection
  
NBTreeNoSplit
  
NBTreeSplit
  
ND
  
NDConditionalEstimator
  
NearestNeighbourSearch
  
NeuralConnection
  
*NeuralMethod*
  
NeuralNetwork
  
NeuralNode
  
NGramTokenizer
  
NNConditionalEstimator
  
NNge
  
Node
  
*NodePlace*
  
NominalPrediction
  
NominalToBinary
  
NominalToBinary
  
NominalToString
  
NonSparseToSparse
  
NormalEstimator
  
NormalizableDistance
  
Normalize
  
Normalize
  
NormalizedPolyKernel
  
NormalMixture
  
NormContinuous
  
NormDiscrete
  
NoSplit
  
NoSupportForMissingValuesException
  
NullStemmer
  
*NumberOfClustersRequestable*
  
NumericCleaner
  
NumericPrediction
  
NumericToBinary
  
NumericToNominal
  
NumericTransform
  
Obfuscate
  
OneR
  
OneRAttributeEval
  
OPTICS
  
OPTICS\_Visualizer
  
Optimization
  
Option
  
*OptionHandler*
  
OptionHandlerJavadoc
  
OrdinalClassClassifier
  
OutputFormatDialog
  
OutputLogger
  
OutputLogger.OutputPrintStream
  
OutputZipper
  
PaceMatrix
  
PaceRegression
  
PairedCorrectedTTester
  
PairedStats
  
PairedStatsCorrected
  
PairedTTester
  
ParentSet
  
Parser
  
Parser
  
PART
  
PartitionedMultiFilter
  
PerformanceStats
  
PKIDiscretize
  
PlaceNode1
  
PlaceNode2
  
Plot2D
  
*Plot2DCompanion*
  
PlotData2D
  
PLSClassifier
  
PLSFilter
  
PMMLClassifier
  
PMMLFactory
  
*PMMLModel*
  
PMMLUtils
  
PNGWriter
  
PointsClosestToFurthestChildren
  
PoissonEstimator
  
PolyKernel
  
PostscriptGraphics
  
PostscriptWriter
  
PotentialClassIgnorer
  
PrecomputedKernelMatrixKernel
  
PreConstructedLinearModel
  
Predicate
  
*Prediction*
  
PredictionAppender
  
PredictionAppenderBeanInfo
  
PredictionAppenderCustomizer
  
PredictionNode
  
PredictiveApriori
  
PreprocessPanel
  
PrincipalComponents
  
PrincipalComponents
  
PrintableComponent
  
*PrintableHandler*
  
PrintablePanel
  
Prior
  
PriorEstimation
  
PriorityQueue
  
PriorityQueueElement
  
Prism
  
PropertyDialog
  
PropertyHandler
  
PropertyNode
  
PropertyPanel
  
PropertyPath
  
PropertyPath.Path
  
PropertyPath.PathElement
  
PropertySelectorDialog
  
PropertySheetPanel
  
PropositionalToMultiInstance
  
ProtectedProperties
  
PruneableClassifierTree
  
PruneableDecList
  
Puk
  
QRDecomposition
  
QueryExecuteEvent
  
*QueryExecuteListener*
  
QueryPanel
  
Queue
  
RacedIncrementalLogitBoost
  
RaceSearch
  
RandomCommittee
  
RandomForest
  
*Randomizable*
  
RandomizableClassifier
  
RandomizableClusterer
  
RandomizableDensityBasedClusterer
  
RandomizableIteratedSingleClassifierEnhancer
  
RandomizableMultipleClassifiersCombiner
  
RandomizableSingleClassifierEnhancer
  
RandomizableSingleClustererEnhancer
  
Randomize
  
RandomProjection
  
RandomRBF
  
RandomSearch
  
RandomSplitResultProducer
  
RandomSubset
  
RandomSubSpace
  
RandomTree
  
RandomVariates
  
Range
  
*RankedOutputSearch*
  
Ranker
  
RankSearch
  
RBFKernel
  
RBFNetwork
  
RDG1
  
ReferenceInstances
  
RegOptimizer
  
Regression
  
RegressionByDiscretization
  
RegressionGenerator
  
RegressionSplitEvaluator
  
RegSMO
  
RegSMOImproved
  
RELAGGS
  
RelationalLocator
  
ReliefFAttributeEval
  
RemoteBoundaryVisualizerSubTask
  
RemoteEngine
  
RemoteExperiment
  
RemoteExperimentEvent
  
*RemoteExperimentListener*
  
RemoteExperimentSubTask
  
RemoteResult
  
Remove
  
RemoveFolds
  
RemoveFrequentValues
  
RemoveMisclassified
  
RemovePercentage
  
RemoveRange
  
RemoveType
  
RemoveUseless
  
RemoveWithValues
  
Reorder
  
RepeatedHillClimber
  
RepeatedHillClimber
  
ReplaceMissingValues
  
REPTree
  
Resample
  
Resample
  
ReservoirSample
  
ResidualModelSelection
  
ResidualSplit
  
ResultChangedEvent
  
*ResultChangedListener*
  
ResultHistoryPanel
  
ResultHistoryPanel.RKeyAdapter
  
ResultHistoryPanel.RMouseAdapter
  
*ResultListener*
  
ResultMatrix
  
ResultMatrixCSV
  
ResultMatrixGnuPlot
  
ResultMatrixHTML
  
ResultMatrixLatex
  
ResultMatrixPlainText
  
ResultMatrixSignificance
  
ResultPanel
  
*ResultProducer*
  
ResultSetHelper
  
ResultSetTable
  
ResultSetTableCellRenderer
  
ResultSetTableModel
  
ResultsPanel
  
ResultVectorTableModel
  
*RevisionHandler*
  
RevisionUtils
  
RevisionUtils.Type
  
Ridor
  
RotationForest
  
Rule
  
Rule
  
Rule
  
RuleGeneration
  
RuleItem
  
RuleNode
  
RuleStats
  
RunNumberPanel
  
RunPanel
  
SaveBuffer
  
*Saver*
  
Saver
  
SaverBeanInfo
  
SaverCustomizer
  
Scanner
  
Scanner
  
ScatterPlotMatrix
  
ScatterPlotMatrixBeanInfo
  
ScatterSearchV1
  
*Scoreable*
  
SearchAlgorithm
  
SelectedTag
  
SelectedTagEditor
  
Sequence
  
SequentialDatabase
  
SERFileFilter
  
*SerialInstanceListener*
  
SerializationHelper
  
SerializedClassifier
  
SerializedInstancesLoader
  
SerializedInstancesSaver
  
SerializedModelSaver
  
SerializedModelSaverBeanInfo
  
SerializedModelSaverCustomizer
  
SerializedObject
  
SerialUIDChanger
  
SERObject
  
SetInstancesPanel
  
SetupModePanel
  
SetupPanel
  
sIB
  
SigmoidUnit
  
SimpleBatchFilter
  
SimpleCart
  
SimpleCLI
  
SimpleCLIPanel
  
SimpleCLIPanel.CommandlineCompletion
  
SimpleDateFormatEditor
  
SimpleEstimator
  
SimpleFilter
  
SimpleKMeans
  
SimpleLinearRegression
  
SimpleLinkedList
  
SimpleLogistic
  
SimpleMI
  
SimpleSetupPanel
  
SimpleStreamFilter
  
SimulatedAnnealing
  
SimulatedAnnealing
  
SingleAssociatorEnhancer
  
SingleClassifierEnhancer
  
SingleClustererEnhancer
  
SingleIndex
  
SingularValueDecomposition
  
SlidingMidPointOfWidestSide
  
SMO
  
SMOreg
  
SMOset
  
SMOTE
  
SnowballStemmer
  
SortedTableModel
  
SortedTableModel.SortContainer
  
*Sourcable*
  
*Sourcable*
  
SparseInstance
  
SparseToNonSparse
  
SpecialFunctions
  
SPegasos
  
SplashWindow
  
SplitCriterion
  
*SplitEvaluate*
  
*SplitEvaluator*
  
Splitter
  
SpreadSubsample
  
SqlViewer
  
SqlViewerDialog
  
Stack
  
Stacking
  
StackingC
  
Standardize
  
*Startable*
  
*StartSetHandler*
  
*StartUpListener*
  
Statistics
  
Stats
  
Stats
  
*Stemmer*
  
Stemming
  
Stopwords
  
StratifiedRemoveFolds
  
*StreamableFilter*
  
StringKernel
  
StringLocator
  
StringToNominal
  
StringToWordVector
  
StripChart
  
StripChartBeanInfo
  
StripChartCustomizer
  
*StructureProducer*
  
SubsetByExpression
  
*SubsetEvaluator*
  
SubsetSizeForwardSelection
  
SubspaceCluster
  
SubspaceClusterDefinition
  
*Summarizable*
  
*SupervisedFilter*
  
SVMAttributeEval
  
SVMLightLoader
  
SVMLightSaver
  
SwapValues
  
*sym*
  
*sym*
  
SymmetricalUncertAttributeEval
  
SysErrLog
  
SystemInfo
  
TabuSearch
  
TabuSearch
  
Tag
  
TAN
  
TAN
  
TargetMetaInfo
  
*Task*
  
*TaskLogger*
  
TaskStatusInfo
  
TechnicalInformation
  
TechnicalInformation.Field
  
TechnicalInformation.Type
  
*TechnicalInformationHandler*
  
TechnicalInformationHandlerJavadoc
  
Tee
  
Tertius
  
Test
  
*Tester*
  
TestInstances
  
TestSetEvent
  
*TestSetListener*
  
TestSetMaker
  
TestSetMakerBeanInfo
  
*TestSetProducer*
  
TextDirectoryLoader
  
TextEvent
  
*TextListener*
  
TextViewer
  
TextViewerBeanInfo
  
ThresholdCurve
  
ThresholdDataEvent
  
*ThresholdDataListener*
  
ThresholdSelector
  
ThresholdVisualizePanel
  
TimeSeriesDelta
  
TimeSeriesTranslate
  
Tokenizer
  
TopDownConstructor
  
TrainingSetEvent
  
*TrainingSetListener*
  
TrainingSetMaker
  
TrainingSetMakerBeanInfo
  
*TrainingSetProducer*
  
TrainTestSplitMaker
  
TrainTestSplitMakerBeanInfo
  
TrainTestSplitMakerCustomizer
  
TreeBuild
  
TreeDisplayEvent
  
*TreeDisplayListener*
  
TreePerformanceStats
  
TreeVisualizer
  
Trie
  
Trie.TrieIterator
  
Trie.TrieNode
  
TwoClassStats
  
TwoWayNominalSplit
  
TwoWayNumericSplit
  
UnassignedClassException
  
UnassignedDatasetException
  
*Undoable*
  
UnsupervisedAttributeEvaluator
  
*UnsupervisedFilter*
  
UnsupervisedSubsetEvaluator
  
UnsupportedAttributeTypeException
  
UnsupportedClassTypeException
  
*UpdateableClassifier*
  
*UpdateableClusterer*
  
UpdateQueue
  
UpdateQueueElement
  
*URLSourcedLoader*
  
UserClassifier
  
*UserRequestAcceptor*
  
Utils
  
Values
  
VaryNode
  
Version
  
VFI
  
ViewerDialog
  
*Visible*
  
VisualizableErrorEvent
  
*VisualizableErrorListener*
  
VisualizePanel
  
VisualizePanel
  
VisualizePanelEvent
  
*VisualizePanelListener*
  
*VisualizePlugin*
  
VisualizeUtils
  
Vote
  
VotedPerceptron
  
WAODE
  
Wavelet
  
*WeightedInstancesHandler*
  
WekaException
  
WekaTaskMonitor
  
*WekaWrapper*
  
Winnow
  
WordTokenizer
  
WrapperSubsetEval
  
XMeans
  
XMLBasicSerialization
  
XMLBeans
  
XMLClassifier
  
XMLDocument
  
XMLExperiment
  
XMLInstances
  
XMLOptions
  
XMLSerialization
  
XMLSerializationMethodHandler
  
XRFFLoader
  
XRFFSaver
  
XStream
  
YongSplitInfo
  
ZeroR
